# An index of multiple deprivation in Sweden: measuring area-level socio-economic inequalities

**DOI:** 10.1093/eurpub/ckaf138

**Published:** 2025-08-29

**Authors:** Lode van der Velde, Ahmed Nabil Shabaan, Mathias Mattsson, Theo Bodin, Terje A Eikemo, Stefan Swartling Peterson, Anna-Karin Danielsson, Emilie E Agardh

**Affiliations:** Department of Global Public Health, Karolinska Institutet, Stockholm, Sweden; Department of Global Public Health, Karolinska Institutet, Stockholm, Sweden; Department of Global Public Health, Karolinska Institutet, Stockholm, Sweden; Institute of Environmental Medicine, Karolinska Institutet, Stockholm, Sweden; Centre for Global Health Inequalities Research (CHAIN), Department of Sociology and Political Science, Norwegian University of Science and Technology (NTNU), Trondheim, Norway; Department of Global Public Health, Karolinska Institutet, Stockholm, Sweden; Makerere School of Public Health, Makerere University, Kampala, Uganda; Department of Global Public Health, Karolinska Institutet, Stockholm, Sweden; Department of Global Public Health, Karolinska Institutet, Stockholm, Sweden

## Abstract

Area-level measures of deprivation in Sweden often rely on limited socio-economic indicators, such as income or education. To address this, we developed the Index of Multiple Deprivation in Sweden (IMDIS) to capture a multitude of explanatory factors for socio-economic inequalities and the distribution across small areas in Sweden. The IMDIS is a compositional index constructed for small areas in Sweden in 2015 and combines 15 indicators across 4 domains (Housing, Employment, Income and Capital, and Education) into an overall deprivation score. Indicators were selected and spatially smoothed to mitigate the effect of small numbers and increase robustness. Domains were constructed using a weighted average of underlying indicators, allowing detailed examination of the significance each domain or indicator has in small areas, and were further combined using explicit weights. All areas were subsequently ranked from the 1^st^ least to 5984^th^ most deprived area. For each area, we generated three key outputs: a score, a rank, and assignment to a deprivation decile. The IMDIS showed high internal consistency and revealed stark geographic inequalities in deprivation. The most deprived areas were concentrated in urban regions, particularly Stockholm, Gothenburg, and Malmö. Housing deprivation was more prominent in urban areas, while educational deprivation was more prevalent in rural and peripheral regions. The IMDIS offers a comprehensive measure of multiple deprivation at the small-area level in Sweden. Its domains and indicators can be used individually or combined to identify inequalities in vulnerable areas and explore geographic patterns, supporting a deeper understanding of social disparities.

## Introduction

Research across multiple disciplines has consistently confirmed that where you live matters for a range of social and health-related outcomes [1, 2]. Although individual determinants often have a larger effect, the characteristics of a place continue to influence people’s experiences and opportunities, even after controlling for individual-level factors [3]. A concept that has particularly been concerned with place is multiple deprivation. While also measurable at the individual level [4], multiple deprivation is traditionally measured at the area level as it is in these places that multiple adversities often converge [5]. Area-level deprivation measures enable comparisons between places and can be used to identify patterns of inequality [6]. The existence of these composite measurements is key in identifying the most vulnerable and disadvantaged areas and to guide local authorities in allocating resources aimed at reducing inequalities [7].

Research on area-level effects has traditionally emphasized compositional aspects of socio-economic position (SEP) [8], often focusing on single or a narrow set of indicators, such as education or income [9]. Failing to recognize the multidimensionality of SEP, which highlights the complex interplay of various factors affecting an individual’s or group’s social and economic standing, limits our understanding of its relationship to various life outcomes, such as health [10]. Additionally, SEP operates at different levels [11] and recognition of the significance of higher-level socio-economic contexts, such as the community or neighbourhood, in shaping inequalities in individual life outcomes is growing [12]. Overlooking the range and scale of socio-economic dimensions can obscure the pathways through which disparities in various life outcomes emerge. In contrast, the concept of multiple deprivation incorporates the multitude of explanatory models for socio-economic inequalities.

Multiple deprivation as an ecological measure was proposed by Peter Townsend and emphasizes the accumulation of a lack of resources required to participate in and adhere to a society’s standard of living [13]. Where the traditional concept of ‘poverty’ focuses primarily on the lack of financial resources [14], multiple deprivation extends to a broader set of material and social conditions and considers an individual’s or group’s relative standing in society. Someone can therefore live above an absolute ‘poverty line’ but still experience a wide range of deprivation states. At the area level, narrow poverty measures can overlook key disadvantages, making some areas seem better off than they are.

In recent years, the development of indices of multiple deprivation (IMDs) at both national and local levels has expanded considerably. Since the first IMDs in the 1970s [15], developments in data collection and availability have opened the door for exhaustive sets of indicators covering the wide range of people’s living environments. Additionally, methods for small-area estimation and the creation of composite indices have significantly improved over the past decades, allowing for more precise insights into multiple deprivation [6]. The English Index of Multiple Deprivation (E-IMD) [6] stands as a comprehensive and widely adopted example, with similar indices subsequently developed in countries such as Scotland [16], New Zealand [17], and Belgium [18].

In Sweden, several examples exist of area-based SEP and deprivation measures [19–21]. Some studies utilized a single indicator, such as income [22], while a multitude of studies have used a composite index based on four indicators (low educational level, low income, unemployment, and receiving social benefits) [23–25]. Despite the widespread use of the four-indicator measure, the rationale for the selection of these specific indicators in their relationship to deprivation appears to be lacking [24]. Moreover, the small areas used in these studies, referred to as SAMS (Small Areas for Market Statistics), were replaced by the DeSO division (Demograsfiska Statistikområden [in Swedish]) in 2018 to better capture homogeneous populations [26] and identify socio-economic conditions over time across Sweden [27] while allowing for aggregation into policy-relevant regions. Strömberg *et al.* [28] recently constructed a four-indicator IMD using open-source data that is based on DeSO areas. Nevertheless, no comprehensive small-area IMD that explores a broad range of deprivation aspects and incorporates recent methodological innovations currently exists in Sweden.

We therefore aimed to develop a comprehensive measure of multiple deprivation at a small-area level, the Index of Multiple Deprivation in Sweden (IMDIS), which captures the multidimensional distribution of deprivation across Sweden. Specifically, our aim was to construct an index that quantifies overall deprivation while also capturing the contributions of individual features. The goal of the IMDIS is to serve as a tool for identifying key indicators of inequality in vulnerable areas and for exploring their relationship to various life outcomes.

## Methods

The construction of the IMDIS was guided by the methodologies developed for the E-IMD and the stages described by Allik *et al.* [29], which are roughly divided into (1) data selection and geographic unit, (2) selection of deprivation domains and indicators, and (3) construction of the index using weights.

### Data selection and geographic unit

The IMDIS is based on DeSO administrative areas, developed by Statistics Sweden, as geographic unit. The DeSO areas are primarily based on population size, with considerable variations in population density across the country, and consider the country’s topography. Integrated within the DeSO classification is a division according to the level of urbanization (rural, peripheral, and urban) and the boundaries of each area adhere to higher-level administrative units such as election districts, municipalities and regions [27].

The IMDIS was developed for the entire Swedish population in 2015 with a registered address, covering 9 834 054 individuals. The residential address of all registered inhabitants was geocoded to a specific DeSO area, resulting in a total of 5984 DeSO areas with a population size ranging from 566 to 2724 inhabitants (mean: 1646.23, SD: 401.52). The average age across DeSO areas in 2015 was 40.69, with younger populations found in urban areas (Supplementary Table S2). Not all individuals could be geocoded to a specific area (*N *= 16 820). This occurred in cases where people had moved abroad without deregistering from Sweden or were incorrectly registered at an address for which an investigation by the Swedish Tax Agency was active.

### Selection of deprivation domains and indicators

The selection of deprivation domains and related indicators was made *a priori*, based on existing literature on multiple deprivation in Sweden [25, 28] and on established indices in other high-income countries [6, 16]. Selection of specific indicators was further guided by evidence for a relative deprivation gradient, data availability, coverage at the small-area unit, and relevance of the indicator in the Swedish context.

**Figure 1.**
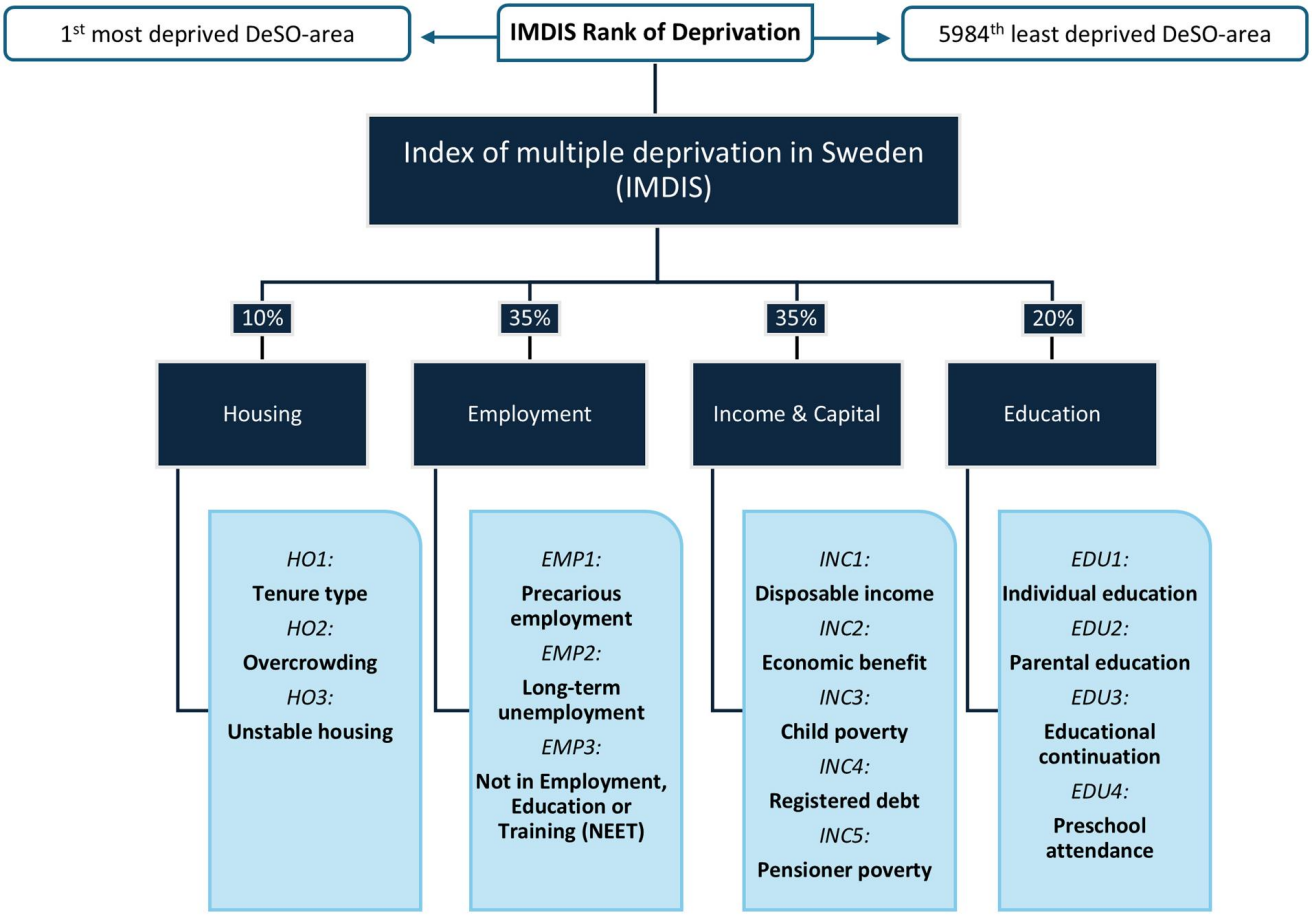
Overview of the IMDIS, domains, and indicators as well as weights used for each domain.

A total of four domains were selected, Housing, Employment, Income and Capital, and Education, covering 15 indicators of deprivation (Fig. 1). All indicators were presented as proportions of a population experiencing deprivation within each DeSO area. The Housing domain covers poor and insecure housing conditions. Overcrowding in this domain was imputed from a higher-level unit called Regional Statistical area (RegSO, which ranges from 567 to 21 463 inhabitants), due to a lack of data on the DeSO-level, assigning each DeSO the value of its corresponding RegSO. The Employment domain considers the proportion of the working-age population not in active labour or experiencing precarious employment conditions. The Income and Capital domain focuses on deprivation because of low-income or registered debt. Included in this domain is income from work, social benefit transfers, and income from capital. Finally, the Education domain comprises deprivation of educational opportunities and attainment.

Within the domains, double counting of individuals was minimized where possible by using age groups and topical distinctions, to avoid overestimating the effect in each domain. For more details on specific domains and indicators, see the Supplementary Material.

### Data sources

Most indicators in the index are based on pseudonymized individual-level data from the Register of the total population, the Longitudinal integrated database for health insurance and labour market studies (LISA), and the Geography database, all held by Statistics Sweden. Data on overcrowding and preschool attendance were obtained from the Swedish National Board of Housing, Building and Planning (Boverket) [21], tenancy form was collected using open-source data from Statistics Sweden [30], and data on registered debt comes from the Enforcement Authority (Kronofogden). For the estimation of the population in precarious employment, the Swedish Job-Exposure Matrix on low employment quality (SweJEM) [31] was used in combination with individual-level register data on occupation from LISA. All data refer to 2015, though multi-year data were used for some indicators to increase counts in small areas (see the Supplementary Material).

### Indicator smoothing

To account for small numbers in some DeSO areas, together with the effect of year-to-year variations in these small numbers and the large standard errors that come with that, a spatial empirical Bayesian smoothing technique, also referred to as shrinkage, was applied [6]. Indicator estimates at the DeSO level were shrunk by ‘linking’ them to a more reliable and robust estimate from a higher geography. This approach not only addresses small sample sizes and increases overall robustness of the data but also considers the at times arbitrary boundaries of the DeSO classification and the spillover effects of indicators across areas.

The selection of the higher-level unit is based on the idea that spatial proximity influences relatedness, suggesting that a reliable estimate should account for all nearby features. We constructed higher-level input areas for each individual DeSO-area based on the first-order queen contiguity principle. When applying this procedure, DeSO areas that constitute an island (10 in total) were manually assigned a higher-level input area using a *k*-nearest neighbours approach, with *k* varying between 1 and 5. Both procedures were performed using QGIS (3.36.2 Maidenhead).

After the shrinkage procedure was applied, which estimates a specific deprivation indicator in a DeSO area using the weighted combination of its own value and the values found for its direct neighbours, all areas retained non-zero estimates. Shrinkage was applied to all indicators except for overcrowding, which was imputed from a higher-level unit from the outset. For more information on the specific procedure, see the technical report for the E-IMD [6].

### Construction of the index using weights

All shrunken indicators were combined into their respective domains using techniques commonly used in IMD development [6, 17]. First, the indicators were made comparable by ranking and transforming them to a normal distribution with a mean of 0 and standard deviation of 1.

For the three domains, Education, Income and Capital, and Employment, regularized common factor analysis (RCFA) with maximum likelihood (ML) was chosen as a method to combine indicators into domains. Confirmatory factor analysis (FA) is particularly suitable for reducing indicators into latent constructs, such as multiple deprivation. Moreover, RCFA effectively handles multicollinearity and addresses Heywood cases, which might produce improper solutions in common factor analysis such as negative unique variances [32]. The RCFA procedure was performed in R version 4.3.3 using the *fareg* function in the *fungible* package. For combining indicators in the housing domain, equal weights were used as one of the indicators (overcrowding) was measured using a higher level (RegSO) estimate, which causes the variability at the DeSO level to not be properly discernible in a FA.

To adhere to the cumulative concept of multiple deprivation and prevent high scores in one domain from completely cancelling out low scores in another when combining domain scores, an exponential transformation of the ranked domain scores was applied (see Supplementary Fig. S1). Additionally, by performing the exponential transformation procedure, greater emphasis is placed on the more deprived areas rather than the least deprived ones [6].

Mirroring the weighting methods proposed by Noble *et al.* [33], six weighting structures were chosen and evaluated for their performance in internal consistency tests using Cronbach’s α and Spearman and Pearson correlation tests against the IMD developed by Strömberg *et al.* [28]. The weight structures included were (1) equal weights, (2) equal weights except for housing, (3) weights derived from and mirroring the distribution of weights in other developed indices [6, 16, 17], (4) weights extracted from a ML FA, (5) weights extracted from a principal component analysis (PCA), and (6) weights used by Strömberg *et al.* [28] (Table 1).

**Table 1.** Weight structures, weights, and outcomes for correlation and internal consistency tests

Weighting approach	Weights (EDU, INC, EMP, HO)	Spearman (Strömberg IMD)	Pearson (Strömberg IMD)	Internal consistency (Cronbach’s α)
1. Equal	.25, .25, .25, .25	0.940*	0.940*	0.869
2. Equal (excl. HO)	.30, .30, .30, .10	0.913*	0.913*	0.857
3. Literature	.20, .35, .35, .10	0.921*	0.924*	0.845
4. ML FA	.22, .30, .27, .21	0.939*	0.939*	0.875
5. PCA	.23, .29, .28, .20	0.938*	0.937*	0.876
6. Strömberg IMD	.08, .35, .32, .25	0.940*	0.944*	0.836

* *P < *0.05.

All weighting schemes showed a high internal consistency of > .8, while not going over .9, which can indicate redundant indicators in the index. Correlation tests showed furthermore strong positive relationships for all weighting structures. Given the satisfactory results across all alternatives, we choose to incorporate the weights based on existing literature (alternative 3) to best reflect the differential contribution of the domains to multiple deprivation. The final, overall IMDIS-score (IMDIS_O_) was calculated based on these weights and subsequently a relative ranking was created from the 1^st^ most deprived area to the 5984^th^ least deprived area. All results were calculated using population-weighted deciles, each representing an equal share of the population.

### Spearman correlation tests

We explored the strength and direction of the relationship between the ranked IMDIS_O_, domains, and individual indicators using Spearman’s rank correlation coefficient.

All procedures, unless stated otherwise, were performed using STATA (release 18, StataCorp, College Station, TX, USA). Geographic visualizations were made using QGIS (3.36.2 Maidenhead).

### Ethics

Ethical approval was granted for the work in this study by the Swedish Ethical Review Authority, under Dnr 2021-00657.

### Availability of results

The IMDIS_O_ ranking of DeSO areas, as well as domain and indicator rankings, can be found at https://github.com/IMDIS/IMDIS2015.

## Results

**Figure 2.**
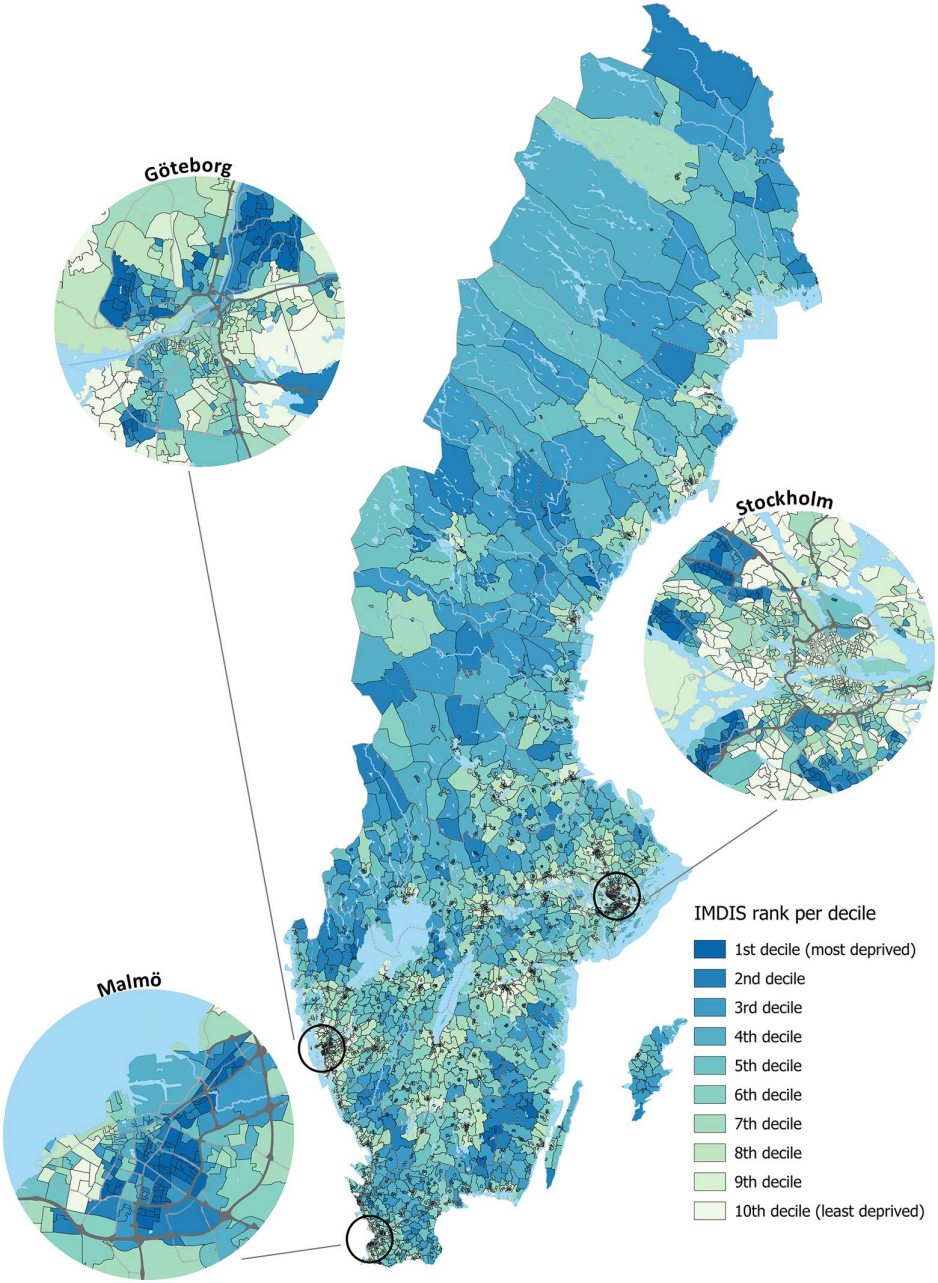
Spatial distribution of IMDIS ranks in deciles each representing a level of deprivation across Sweden, and in the three most populous cities (Stockholm, Gothenburg, and Malmö) in 2015.

The IMDIS was calculated for the 5984 administrative DeSO areas in Sweden in 2015 (Fig. 2), which showed a clear deprivation gradient for IMDIS_O_ scores (Table 2). In a ranked order, the 1^st^ most deprived area was located in Trollhättan municipality and the 5984^th^ least deprived area was in Ekerö municipality.

**Table 2.** Mean domain and total scores by level of urbanization and population weighted deciles, and average age by deciles of IMDIS_O_

			Deciles of deprivation									
			Most deprived									Least deprived
Domain	Urbanization	Total	1^st^	2^nd^	3^rd^	4^th^	5^th^	6^th^	7^th^	8^th^	9^th^	10^th^
IMDIS_O_		5.43 (5984)	16.02 (538)	10.37 (591)	7.68 (614)	5.92 (622)	4.67 (623)	3.67 (611)	2.8 (613)	2.09 (605)	1.41 (600)	0.64 (567)
(mean (*n*))	Peripheral	5.18 (580)	13.92 (34)	10.31 (77)	7.74 (65)	6.01 (64)	4.66 (52)	3.65 (58)	2.83 (59)	2.07 (53)	1.41 (53)	0.66 (65)
	Rural	4.66 (1080)	13 (4)	9.9 (54)	7.61 (138)	5.88 (183)	4.67 (201)	3.66 (181)	2.79 (146)	2.11 (98)	1.46 (65)	0.75 (10)
	Urban	5.65 (4324)	16.19 (500)	10.43 (460)	7.7 (411)	5.93 (375)	4.68 (370)	3.68 (372)	2.79 (408)	2.08 (454)	1.4 (482)	0.63 (492)
*Education domain* (mean (*n*))		21.71 (5984)	57.34 (538)	39.9 (591)	32.28 (614)	25.29 (622)	20.36 (623)	15.38 (611)	11.68 (613)	8.64 (605)	6.11 (600)	3.21 (567)
	Peripheral	28.27 (580)	75.76 (34)	53.84 (77)	42.65 (65)	33.74 (64)	25.74 (52)	20.37 (58)	16.16 (59)	10.48 (53)	8.52 (53)	4.04 (65)
	Rural	26.37 (1080)	65.1 (4)	54.41 (54)	40.4 (138)	33.4 (183)	26.99 (201)	20.71 (181)	16.72 (146)	13.42 (98)	9.31 (65)	5.49 (10)
	Urban	19.67 (4324)	56.02 (500)	35.86 (460)	27.92 (411)	19.88 (375)	16.01 (370)	12.01 (372)	9.23 (408)	7.4 (454)	5.41 (482)	3.05 (492)
*Income & Capital domain* (mean (*n*))		21.71 (5984)	67.09 (538)	43.47 (591)	31.92 (614)	24.15 (622)	18.36 (623)	13.93 (611)	10.15 (613)	6.81 (605)	3.98 (600)	1.5 (567)
	Peripheral	17.40 (580)	47.14 (34)	37.04 (77)	27.35 (65)	20.16 (64)	15.52 (52)	11.08 (58)	8.25 (59)	6.18 (53)	3.71 (53)	1.71 (65)
	Rural	18.51 (1080)	48.28 (4)	37.63 (54)	30.38 (138)	23.71 (183)	19.05 (201)	14.47 (181)	10.91 (146)	8.14 (98)	5.72 (65)	2.42 (10)
	Urban	23.09 (4324)	68.6 (500)	45.23 (460)	33.15 (411)	25.05 (375)	18.39 (370)	14.1 (372)	10.15 (408)	6.6 (454)	3.78 (482)	1.45 (492)
*Employment domain* (mean (*n*))		21.71 (5984)	67.2 (538)	43.26 (591)	30.99 (614)	23.24 (622)	18.24 (623)	13.93 (611)	9.87 (613)	7.42 (605)	4.92 (600)	2.4 (567)
	Peripheral	22.34 (580)	61.87 (34)	45.33 (77)	32.43 (65)	25.38 (64)	19.59 (52)	16.09 (58)	11.93 (59)	9.42 (53)	5.77 (53)	2.64 (65)
	Rural	18.00 (1080)	58.54 (4)	42.38 (54)	31.88 (138)	22.89 (183)	17.44 (201)	13.64 (181)	9.76 (146)	6.68 (98)	3.9 (65)	2.04 (10)
	Urban	22.56 (4324)	67.64 (500)	43.02 (460)	30.45 (411)	23.05 (375)	18.48 (370)	13.74 (372)	9.62 (408)	7.35 (454)	4.97 (482)	2.38 (492)
*Housing domain* (mean (*n*))		21.71 (5984)	56.19 (538)	31.31 (591)	22.63 (614)	20.52 (622)	18.11 (623)	18.47 (611)	18.38 (613)	16.37 (605)	12.91 (600)	5.4 (567)
	Peripheral	11.48 (580)	23.57 (34)	16.28 (77)	15.12 (65)	13.66 (64)	11.95 (52)	10.24 (58)	10.15 (59)	7.27 (53)	6.34 (53)	3.23 (65)
	Rural	5.84 (1080)	15.87 (4)	7.28 (54)	5.8 (138)	5.48 (183)	5.02 (201)	6.53 (181)	5.92 (146)	5.9 (98)	5.91 (65)	3.44 (10)
	Urban	27.05 (4324)	58.74 (500)	36.65 (460)	29.46 (411)	29.03 (375)	26.08 (370)	25.56 (372)	24.03 (408)	19.69 (454)	14.57 (482)	5.72 (492)
Average age (mean (95% CI))		40.96 (40.84–41.08)	37.02 (36.61–37.43)	41.49 (41.08–41.9)	42.72 (42.35–43.09)	42.51 (42.16–42.86)	42.68 (42.35–43.01)	41.77 (41.44–42.1)	41.28 (40.97–41.59)	41.05 (40.72–41.38)	39.98 (39.67–40.29)	38.41 (38.11–38.71)

All domains showed a steady gradient in deprivation, with scores progressively increasing from the least deprived (1^st^ decile) to the most deprived areas (10^th^ decile) (Table 2). The most deprived areas were concentrated in urban areas, with 30% of all areas in Malmö found in the first deprivation decile, 18.3% of areas in Gothenburg, and 9.4% of areas in Stockholm. In contrast, the largest share of areas in the 10^th^ decile (least deprived) was found in Gothenburg (13.4%), followed by Stockholm (10.1%) and Malmö (2.1%). The urban-rural gradient was inconsistent across IMDIS_O_ and domain scores. A higher mean IMDIS_O_ score was demonstrated in urban areas (5.65) compared to peripheral (5.18) and rural areas (4.66) (Table 2). This gradient was also evident in the Housing and Employment domains, whereas it was reversed in the Education domain and showed a different, less consistent pattern in the Income and Capital domain. For domain-specific outcomes, the highest proportion of most deprived areas were in the municipality of Stockholm for Housing (18.7%), Gothenburg for Education (4.6%), and Malmö for Income and Capital (11.8%), and Employment (9.9%).

Considering the age structure in each DeSO, the first IMDIS_O_ decile had an average age of 38.37 (95% CI 38.08-38.66), followed by a steady increase in age until the 8th decile (42.74; 95% CI 42.36–43.12) (Table 2). Similar to the most deprived areas, the population in the least deprived decile tends to be younger (37.21; 95% CI 36.82-37.6).

The IMDIS showed strong positive correlations with most indicators and across all domains, except for the Housing domain (see Supplementary Fig. S2). Positive correlations were also observed between the Employment and Income and Capital domains (ρ  =  0.85, *P *< 0.05), as well as between Employment and Education (ρ  =  0.72, *P *< 0.05). The Housing domain was only weakly but significantly correlated with the Education domain (ρ  =  0.14, *P *< 0.05). All housing indicators were negatively correlated with parental education, educational continuation, and precarious employment, except for tenure type, which was not significantly associated with individual education.

When considering the 21 regions in Sweden, the geographic distribution of deprivation displays similar unequal patterns (Supplementary Figs S3 and S4), where Stockholm county demonstrates the lowest median IMDIS_O_ score (2.65), while Värmland county shows the highest score (5.98).

## Discussion

In this study, we constructed a multidimensional and high-resolution index of deprivation, with the aim to capture the distribution of deprivation at the small-area level across Sweden. Results underlined the uneven geographical distribution of deprivation across levels of urbanization, regions, and age.

Recent attempts to capture deprivation in a composite index included four-indicator measures [23–25] or single indicator proxies for deprivation [22]. Another study employed a set of indicators from the first UK IMD [34], in spite of the debatable relevance of factors such as car-ownership for identifying disadvantage in the Swedish context [35]. Some widely used indices were created for specific purposes including identifying SEP in relation to crime prevention [20], health [24], and for monitoring segregation [21]. We attempted to avoid ambiguous definitions of indicators by building on relevant literature on multiple deprivation in the Swedish context [25, 28] as well as literature regarding a relative socio-economic gradient for indicators (see the Supplementary Material). To our knowledge, the IMDIS is the first composite index to comprise a broader range of deprivation attributing indicators, using both register data and data from public sources, at a small-area level in Sweden.

Strömberg *et al.* [28] presented a four-indicator IMD at the DeSO area level, using common factor analysis to combine indicators. We followed their work in that we used the DeSO division as a geographic unit to better represent homogenous populations compared to previous IMDs in Sweden, and built on the same domains (Education, Income, Employment, and Housing). However, the significance of indicators can vary between different areas, and a more detailed understanding of the specific elements in each domain will help identify entry points for policy and targeted action. Therefore, we broadened the underlying factors that influence each domain.

### Strengths and limitations

There are some limitations with regards to the methodological choices made when creating the IMDIS. First, double counting of individuals within domains to avoid overestimating the level of (domain) deprivation can occur. We limited this primarily through age clustering in specific indicators. However, as multiple deprivation implies, highly deprived areas are characterized by multiple correlated adversities. In line with earlier developed indices [6], the assumption is therefore that double counting is not completely avoidable.

Second, exponential transformation of the domain scores was implemented to abet domain scores on opposite sides of the distribution cancelling each other out and to highlight the most deprived areas more so than the least deprived. Consequently, the index is less capable of identifying differences on the well-off side of the deprivation distribution. While this is important to consider when carrying out inequalities research, any interventions are more likely to be effective when targeted at the deprived end of the IMDIS distribution [36].

Third, McCartney *et al.* [37] highlight some limitations of the use of composite indices in inequality research. First, by assigning individuals to the average deprivation score of the area they live in, overall inequalities tend to be forcibly smaller (closer to zero) as a result. Second, using the index to identify deprived individuals should be avoided, as it risks ecological fallacy. It is therefore crucial to view the results of the IMDIS in the context of socio-demographic differences between areas. Moreover, restricting the population to individuals with a registered address may exclude particularly vulnerable groups without one, such as undocumented, or homeless populations. Consequently, any inferences regarding the absolute size of the multiply deprived population in Sweden should be avoided.

In addition, there are broader limitations related to the nature of spatial data and the assumptions underlying area-based research. A foremost critique of place-based research is its reliance on static geographic units, which often fail to capture the dynamic nature of people’s movements across administrative boundaries. It has been shown that administrative boundaries have little relevance in explaining inequalities in, for instance, individual mortality, with household contexts playing a larger role [3]. This challenges the assumption that people primarily experience exposures within their neighbourhood boundaries. To address these limitations, this study considers spill-over or autoregressive spatial effects when constructing indicators, which also reduces bias from the Modifiable Areal Unit Problem [38]. The DeSO-classification is specifically prone to measurement error when viewed as a neighbourhood measure because of its reliance on population size. Although the IMDIS uses a shrinkage procedure, its dependence on DeSO areas risks selection bias and should be interpreted cautiously.

Finally, there are differences across Sweden with regards to the cost of living and purchasing power [39]. The IMDIS is an IMD measured at the national level which skews some of the rankings based on differences between areas. This is maybe most pronounced in indicators using a poverty line. In the disposable income indicator, having an income under the poverty line might make you worse off in areas where the cost of living is higher than the national average compared to areas below it. However, Oudin *et al.* [40] show that the difference in the effect of small-area deprivation is negligible when assessed on a national or regional level. We therefore maintain the same risk-of-deprivation line for the whole of Sweden.

A key strength of the IMDIS is its capacity to quantify overall deprivation while also disentangling the specific contributions of individual domains and indicators, enabling tailored analyses aligned with distinct policy objectives and research questions. Its design also supports aggregation from DeSO units to higher geographic levels, allowing for application at other policy-relevant scales. In developing the IMDIS, we expanded on existing methodologies by addressing uncertainty at the small-area level through a shrinkage technique, and by mitigating ‘cancellation’ effects between domains using exponential transformation. Together, these methodological choices contribute to a more nuanced understanding of deprivation and offer valuable insights for future research on its impact across various life outcomes.

## Conclusion

The IMDIS provides a comprehensive account of multiple deprivation at the small-area level in Sweden, addressing the general lack of precise measures that integrate diverse data sources and recent methodological innovations in MDI development. The domains and indicators within the IMDIS can be used independently or in combination to explore their significance within each small area. We suggest using the IMDIS to identify key indicators of inequality in vulnerable areas across Sweden and to explore geographic patterns across small areas, helping to improve the understanding of these inequalities.

## Supplementary Material

ckaf138_Supplementary_Data

## Data Availability

Due to ethical restrictions, the individual-level data used in this study are not publicly available. However, the data supporting the findings of this study can be obtained from the following sources: Statistics Sweden (microdata and open-source data through https://www.statistikdatabasen.scb.se), Kronofogden, and Boverket (https://www.boverket.se/sv/om-boverket/oppna-data). Key points A comprehensive multiple deprivation index is still lacking in Sweden. This study presents the Index of Multiple Deprivation in Sweden. Deprivation disparities persist across levels of urbanization, regions, and age. The index is proposed as a tool to more precisely identify vulnerable areas.
