# Supplementary material for: An index of multiple deprivation in Sweden: measuring area-level socio-economic inequalities
Source: Eur J Public Health. 2025 Aug 29;35(6):1107–14. doi: 10.1093/eurpub/ckaf138 (PMC12707479; doi:10.1093/eurpub/ckaf138)
Supplement: ckaf138_Supplementary_Data [file ckaf138_supplementary_data.docx]

Contents

[Indicators in the IMDIS 2](#_Toc204204006)

[**Domain: Education** 2](#_Toc204204007)

[**Domain: Income & Capital** 5](#_Toc204204008)

[**Domain: Employment** 9](#_Toc204204009)

[**Domain: Housing** 11](#_Toc204204010)

[Tables and figures 17](#_Toc204204011)

[Figure S1: IMDIS_O_ scores across all 5984 DeSO areas 18](#_Toc204204012)

[Figure S2: Correlation matrix of the IMDIS 19](#_Toc204204013)

[Figure S3: Distribution of IMDIS_O_ scores by 21 regions 20](#_Toc204204014)

[Figure S4: Distribution of domain-scores by region 21](#_Toc204204015)

[Table S1: Mean and total indicators scores, with numbers of DeSO areas, by population weighted deciles 22](#_Toc204204016)

[Table S2: Average age with 95% Confidence intervals by population weighted deciles of IMDIS_O_ and level of urbanization 23](#_Toc204204017)

## Indicators in the IMDIS

Most indicators in the Index of Multiple Deprivation in Sweden (IMDIS) have been constructed using a comprehensive dataset of register linkages, covering both demographic and socio-economic variables. In some cases, data from public or other sources were utilized for the creation of specific deprivation indicators.

*Households*

Households in the IMDIS are based on the definition used by Statistics Sweden (SCB) and refers to all individuals who are registered at the same residence. This differs from the family definition, which apart from the residence factor also considers (familial) ties through a civil union, registered partnership or through parental linkage. There are approximately 4,9 million families and 5,7 million households in Sweden (1). Using households instead of families increases our chances of identifying and including household settings that are not based on the above.

*Changes in DeSO classifications*

From 2018 onwards two DeSO areas (1883A0010 & 1883A0020) are merged into a single area (1883A0030) in the Karlskrona municipality. Considering potential future expansions of the IMDIS to cover multiple years, we applied this merger for the year 2015 as well.

### **Domain: Education**

The education domain measures to what extent educational attainment is missing in the studied population. The body of proof for the educational gradient in life outcomes, such as health, employment status, and criminal behaviours, is strong. In a European context low educational attainment leads to worse health outcomes for all-cause mortality and most specific causes compared to those with high educational attainment (2, 3). Increased educational years have also been shown to improve employment status (4) and protects against later life criminal behaviours (5). On a societal level, increased educational attainment strengthens political participation (6) and social trust (7).

To avoid double counting between indicators in this domain, each variable represents a different period in educational trajectories. EDU4 focuses on preschool education with ages ranging from 1 to 5, EDU2 describes parental education for children under the age of 17, EDU3 captures post-secondary education between ages 16 to 25 and EDU1 represents the overall educational level in the adult (25+) population. Finally, EDU2 represents educational deprivation within household settings.

#### EDU1: Individual educational attainment

- Proportion of people with less than 9 years of formal education

*Evidence*

Individual educational attainment is one of the most prominently used indicators of socio-economic position (8). Many of the positive effects of individual education run through earning potential, or increased income, which offers a range of positive effects on multiple life skills (9). At the same time individual education itself has been shown to influence fertility and reproductive decisions (10) as well as cognitive skills (11). Furthermore, education is a fundamental cause of health and disease that works through attributes such as access to higher income, better neighbourhoods and healthier diets (12). Moreover, higher educational attainment is a reliable predictor for health outcomes as it lies upstream from many other social determinants of health (13).

*Operationalization*

Highest attained education for each individual was classified as follows; low (primary or lower secondary education, ≤ 9 years of study), middle (upper secondary education, corresponding to 10-12 years of study), and high (post-secondary or tertiary education, corresponding to >12 years of study).

*Numerator:* Individuals with less than 9 years of formal education

*Denominator:* Total adult population

*Age limits:* 25-100

*Geography:* DeSO

*Data sources:* Longitudinal integrated database for health insurance and labour market studies (LISA), the Geography database (GDB) & the Register of the total population (RTB)

#### EDU2: Parental education

- Proportion of children in households where the highest education is less than 10 years

of formal education

*Evidence*

Children living in households where the caregivers have low educational attainment face a range of disadvantages. Among the most direct is lower academic achievement (14), language development, and social skills (15). Parental education has been linked to a range of poor health outcomes in children such as child mortality and obesity (16, 17). In Sweden, influence of parental education on child health has also been found on both the area level as well as the individual level (18).

*Operationalization*

All children aged 17 and younger were linked to their respective caregivers (adoptive or biological). Using the classifications for education mentioned under EDU1, highest individual educational attainment between the caregivers was assigned to the children.

*Numerator:* Children in households where the highest education is less than 10 years of formal education

*Denominator:* Total households

*Age limits:* 0-17

*Geography:* DeSO

*Data sources:* LISA, GDB & RTB

#### EDU3: Educational continuation

- Proportion of young adults not continuing in tertiary education after secondary school

*Evidence*

This indicator follows the conventional pathways described under EDU1, such increased opportunities for employment and income (19). Evidence for a positive effect of additional years of schooling on health are primarily found around studies investigating the effect of extended compulsory schooling (20). Moreover, a clear dose-response effect was observed for every additional year of education in relation to, for instance, the risk of mortality (21) and has a protective effect on employment stability (22).

*Operationalization*

After completion of secondary school between the ages of 17 and 21, individuals were followed for 5 consecutive years to record entrance into tertiary education. Receiving any study allowance was used as proxy for active education. Thus, not receiving any allowance at any point within these 5 years was marked as not having continued in education. To increase cases in small-areas, multiple years (2012, 2013, 2014) were used to construct this indicator.

*Numerator:* Individuals not continuing in tertiary education within 5 years after finishing secondary school (gymnasium)

*Denominator:* All individuals who finished secondary school

*Age limits:* 17-21

*Geography:* DeSO

*Data sources:* LISA, GDB & RTB

#### EDU4: Preschool attendance

- Proportion of children not registered in pre-school

*Evidence*

Early-life education, such as pre-school, is not mandatory in Sweden but has developmental advantages compared to children who do not attend pre-school between the ages of 1 and 5 (23). In a Swedish context this has been scarcely studied, however in other Nordic countries with similar pre-school structures, a positive effect on among others mental health (24) and emotional development (25) has been observed. Moreover, pre-school attendance strengthens higher educational attainment at older ages as well (26, 27).

*Operationalization*

Data for this indicator was obtained from the Segregation barometer (Segregationsbarometer in Swedish) (28), which is based on data from The Swedish National Agency for Education (Skolverket in Swedish) and specifies the number of registered children in pre-school in 2015 according to their place of residence at the end of that year. Since the IMDIS focuses on deprived populations, the final indicator was altered to represent those *not* registered in preschool.

*Numerator:* Children not registered in preschool

*Denominator:* Total child population

*Age limits:* 1-5

*Geography:* DeSO

*Data sources:* RTB, GDB & Segregationsbarometer (Swedish National Board of Housing, Building and Planning (Boverket), The Swedish National Agency for Education (Skolverket)

### **Domain: Income & Capital**

The Income & Capital domain covers deprivation because of a lack of income or the burden of debt, and it measures the proportion of the population experiencing this in a specific area. Income deprivation has been shown to predict a range of outcomes on both area and individual levels (29). Low income often intersects with other socio-economic factors such as education and employment, which can perpetuate the cycle of poverty. The Income & Capital domain includes income sources of those in and out of work (salary as well as benefits). INC1, INC3 and INC5 use a relative poverty line as defined by the EU, which is defined as 60% of the median income in a specific population (30).

Double counting between indicators was limited based on topical distinctions and age-groups. However, some overlap is expected between Disposable income (INC1 and INC5) on the one hand and receiving economic benefits (INC2) on the other hand.

#### INC1: Disposable income

- Proportion of people with a disposable income under the poverty line

*Evidence*

The link between individual disposable income and social and health outcomes is well established, showing a consistent gradient in which individuals with higher incomes generally experience better outcomes than those with lower incomes (29). The pathways between income and health run through limited financial means, which can cause stress and limited access to resources (31), but also through its connection to a range of other social determinants of health (32). Ecological effects of income on health have also been found in Sweden, for instance in relation to mortality (33) and ischemic heart disease (34). In addition, disposable income affects the accumulation of material advantages like housing quality (35), as well as social outcomes such as access to social capital (36).

*Operationalization*

This indicator includes disposable income per consumption unit of the household the individual belongs to. It also includes income earned in other Nordic countries. Students have been excluded from both the numerator and denominator.

*Numerator:* Individuals with a disposable income under the poverty line

*Denominator:* Total working-age population

*Age limits:* 18-64

*Geography:* DeSO

*Data sources:* LISA, GDB & RTB

#### INC2: Economic benefits

- Proportion of people receiving economic benefits

*Evidence*

In general, receiving economic benefits can be seen as a marker of low income or poverty, but can also be the consequence of other circumstances (37). This indicator, therefore, follows similar pathways to those outlined for low disposable income in its connection to material, social, and health outcomes. However, not everyone living under the poverty line necessarily receives benefits, which gives an indication of a deprived population facing barriers to accessing public benefits (38).

*Operationalization*

Economic benefits include subsistence allowance or welfare benefits to cover basic living expenses (social bidrag/försörjningsstöd). While paid for by the state, conditions for receiving these benefits are often set by local authorities. Therefore, differences between municipalities exist as to what this benefit covers. However, in general benefits are seen as short-term financial aid for individuals who are facing financial difficulties and who have exhausted all other benefits and options available to them.

*Numerator:* Individuals receiving economic benefits

*Denominator:* Total households

*Age limits:* 18-100

*Geography:* DeSO

*Data sources:* LISA, GDB & RTB

#### INC3: Child poverty

- Proportion of children in households under the poverty line

*Evidence*

The effect of low income can spillover onto children as well, both directly and indirectly, and influences various aspects of their physical, mental, and developmental wellbeing. For instance, financial constraints in a household has been associated with food insecurity and limited access to nutritious food (39). These circumstances have been linked to a range of health outcomes later in life such as diabetes and cardiovascular disease (40). In turn, poor nutrition can also affect growth and cognitive development (41). Children living in poverty are also more likely to be exposed to adverse childhood experiences, which can have lasting impacts on their health, well-being, and social development (42). Financial strain can furthermore increase stress among parents and lead to mental health issues as well as limited parental abilities, which has been shown to negatively impact children’s emotional and psychological wellbeing (43).

*Operationalization*

This indicator includes disposable household income of the household the child belongs to. It also includes income earned in other Nordic countries. Children were linked to their respective household using parental linkages (adoptive or biological). Children of separated parents were assigned the average disposable income between the two parents’ households.

*Numerator:* Children in households where the household disposable income lies under the poverty line

*Denominator:* All children

*Age limits:* 0-17

*Geography:* DeSO

*Data sources:* LISA, Flergenerationsregistret (FGR), GDB & RTB

#### INC4: Registered debt

- Proportion of encounters with The Enforcement Authority (Kronofogden) related to a debt

*Evidence*

Debt is linked to a range of negative outcomes and can create significant barriers to accessing resources that support life improvement. Debt is primarily associated with negative health outcomes through financial stress, which contributes to conditions like hypertension, cardiovascular disease (44), and mental health disorders. The psychological burden of debt is profound and studies have shown that people in debt are more likely to experience mental health disorders such as anxiety and depression (45). At the same time, registered debt can create barriers to quality housing and employment opportunities (46).

*Operationalization*

The number of referrals of private individuals to The Enforcement Authority, which is responsible for debt collection, enforcing court orders, and managing bankruptcies, was obtained at an aggregated postal code level. Since postal area (roughly 10.000) don’t aggregate directly into DeSO area (roughly 6000), a GIS overlay analysis was used to assign postal area’s to DeSO areas. Postal areas were assigned to a DeSO based on the largest surface area found within a DeSO. The polygon file for postal areas was acquired from Postnord (GEPOSIT). The number of years included in this variable was broadened to get to a sufficient number of cases in each area and to strengthen the reliability of the estimate.

*Numerator:* All registrations of debt with The Enforcement Authority (Kronofogden) between the years 2013-2015

*Denominator:* Total adult population between 2013-2015

*Age limits:* 18-100

*Geography:* DeSO

*Data sources:* GDB, Postnord, RTB & The Enforcement Authority

#### INC5: Pensioner poverty

- Proportion of elderly with a disposable income under the poverty line

*Evidence*

Poverty in older adults is associated with many of the same negative outcomes linked to low disposable income, including reduced access to healthcare and housing insecurity (47). However, there are additional consequences that are particularly pronounced in this age group. One of the most significant is the heightened impact of loneliness and social isolation. Poverty in later life amplifies these experiences, which have been strongly linked to poorer physical and mental health, as well as accelerated cognitive decline (48).

*Operationalization*

This indicator includes disposable income per consumption unit of the household the individual belongs to. It also includes income earned in other Nordic countries.

*Numerator:* Individuals over 65 with an income under the poverty line

*Denominator:* Total population over 65

*Age limits:* 65-100

*Geography:* DeSO

*Data sources:* LISA, GDB & RTB

### **Domain: Employment**

The employment domain covers several aspects of being employed as well as the quality of employment. It therefore focuses both on occupation as well as several aspects of being (un)employed. It measures the proportion of the working-age population in a specific area experiencing employment deprivation. The relationship between employment and social and health outcomes is often characterized as bidirectional, with each influencing the other. For instance, poor health decreases the chances of being in employment, and being employed similarly influences health, positively and negatively (49).

Double counting between indicators has been avoided by using age distinctions as well as specific non-overlapping operationalizations for the Precarious employment- and NEET-populations.

#### EMP1: Precarious employment

- Proportion of individuals in precarious employment

*Evidence*

Precarious employment (PE) is a multifaceted concept which identifies and brings together income inadequacy, employment insecurity, and a lack of rights and protections. PE is becoming increasingly prevalent in Sweden and is associated with various negative health outcomes such as cardiovascular disease and stroke (50), and work-related injuries (51). PE furthermore influences social outcomes such as family planning and partnership formation (52), and overall life satisfaction (53).

*Operationalization*

This indicator is constructed using the job-exposure matrix for low employment quality (SweJEM, (54)). The SweJEM is specifically developed for identifying PE in the Swedish population, using occupational codes found in LISA, which describe type of occupation. PE populations were estimated based on the make-up of occupations found among the working-age and employed populations in each DeSO-area. Coverage for the occupational codes in LISA in 2015 has been poorer compared to subsequent years (55), we therefore use occupational codes from LISA 2016.

*Numerator:* Population in precarious employment

*Denominator:* Working age population

*Age limits:* 18-64

*Geography:* DeSO

*Data sources:* RTB, GDB, LISA & SweJEM

#### EMP2: Long-term unemployment

- Proportion of individuals receiving unemployment benefits

*Evidence*

Long-term unemployment has a profound impact on various aspects of an individual’s life, including health, well-being, social participation, and economic stability. Studies indicate that the unemployed are more likely to experience psychological distress, depression and suicidal tendencies (56). Other consequences of unemployment, such as loss of income and social status, can exacerbate these issues. This impact extends to physical health as well, where long-term unemployment raises the risk of developing chronic conditions such a cardiovascular disease and diabetes (57). Prolonged detachment from the labour market often weakens social participation and networks (58) and introduces difficulties re-entering the labour market (59). Over time, these factors can spill over into family and community contexts as well.

*Operationalization*

Unemployment is based on the total number of days an individual has been recorded as unemployed by the Swedish Public Employment Service (Arbetsförmedlingen). An individual needs to be registered as a job seeker and does not require to receive benefits of any kind. Following previous research (60), long term-unemployment is set at 90 days or more.

*Numerator:* Individuals in long-term unemployment

*Denominator:* A working-age population

*Age limits:* 31-64

*Geography:* DeSO

*Data sources:* LISA, GDB & RTB

#### EMP3: Not in Employment, Education or Training (NEET)

- Proportion of young adults not in employment, education or training

*Evidence*

The term NEET (Not in Employment, Education, or Training) covers young individuals who are unemployed and inactive, not participating in any formal or informal education, as well as those experiencing long-term illness, or who are otherwise unable or unavailable to work (61). NEET has been associated with a range of negative mental health outcomes such alcohol use disorders (62), psychiatric disorders (63), anxiety, and depression (64). NEET-Individuals experience similar social disadvantages as those in long-term unemployment, such social isolation and reduced employability (65). On a societal level, large NEET populations can result in lost labour and economic potential and higher public costs (66).

*Operationalization*

NEET populations are identified using an indexed price base amount (prisbasbelopp), established by the Swedish government as the minimum income required to meet basic living needs. Individuals receiving an income below the price base amount, not receiving any study grants, and not being registered for education for more than 60 hours per year are characterized as being NEET. This indicator covers the proportion of young people in NEET in 2015.

*Numerator:* Individuals not in Employment, Education or Training (NEET)

*Denominator:* Young adult population

*Age limits:* 17-30

*Geography:* DeSO

*Data sources:* LISA, GDB & RTB

### **Domain: Housing**

The housing domain measures the proportion of individuals experiencing housing deprivation and covers aspects of housing stability and quality. Housing can influence social and health outcomes through several pathways related to stability, quality and affordability (67). In Sweden, those living in poorer housing conditions, rental housing and those living in overcrowded houses are often also found at the deprived end of other socio-economic factors such as income and education (68).

Double counting between indicators in this domain has primarily been avoided using topical distinctions between indicators.

#### HO1: Tenure type

- Proportion of individuals in rental housing

*Evidence*

Housing conditions, such as tenure type, heating and costs have been linked to faster biological aging. Especially, privately rented housing and housing affected by pollution were predictors for faster aging compared to owning housing outright (69). Moreover, tenure type lies on this pathway as (private) rental houses are often in poorer condition compared to privately owned houses. However, the relatively large social housing sector in Sweden moderates the magnitude of the rental-health association because social housing is generally in good condition. Nonetheless even in Sweden a tenure-health relationship has been found (70). In addition, individuals in rental housing face a higher risk of housing instability and areas with higher proportions of rental housing tended to have higher crime rates (71).

*Operationalization*

The tenure type is based on ownership rather than who occupies the residence. Four distinctions were made between types of housing: rental housing, tenant owned apartments, outright ownership, and other. This indicator describes the proportion of individuals living in rental housing.

*Numerator:* Individuals in rental housing

*Denominator:* Total population

*Age limits:* No age limits

*Geography:* DeSO

*Data sources:* SCB Statistics Sweden

#### HO2: Overcrowding

- Proportion of overcrowded households

*Evidence*

The number of individuals in Sweden who live in overcrowded living spaces has in the past 15 years slowly increased (68). Overcrowding can increase household conflicts and the risk for domestic abuse and antisocial behaviour (72). Children living in crowded houses have been shown to perform worse in school (73). The quality of a home knows a direct pathway between housing and health, which can be attributed to factors such as mould and moisture causing for instance respiratory illnesses (74). Overcrowding (as measured by persons per room) has been found to damage not only physical health (75), but moreover puts a strain on people’s mental health (76).

*Operationalization*

A household is assumed to be overcrowded, according to Boverket’s Norm 2, if a single person household does not have a separate bedroom apart from a living room and kitchen. Any additional person needs their own bedroom. Two children can share a bedroom up to the age of 11. In cases where data on the number of rooms in a home is missing, the average living area per person is used to determine overcrowding, where less than 20 m^2^ per person is considered overcrowded. Single person households without children are not considered overcrowded. Data for this indicator was only publicly available at the RegSO level. DeSO estimates were derived by imputing values from the corresponding RegSO in which each DeSO was located.

*Numerator:* Overcrowded households according to norm 2

*Denominator:* Total households

*Age limits:* No age limits

*Geography:* RegSO

*Data sources:* Boverket/Delegation mot segregation (Delmos)(73)

#### HO3: Unstable housing

- Proportion of individuals moving more than twice in the past 3 years

*Evidence*

A continuously changing population hinders the ability for communities to establish strong social bonds within specific areas (77), which in turn may contribute to negative outcomes such as higher crime rates (78). On an individual level, lacking stable housing has a negative effect on both mental and physical health. Where homelessness in and of itself has detrimental effects on health (79), the threat of homelessness and the instability of housing overall is associated with psychological health such as depression, but also risk behaviours such as increased alcohol use (80).

*Operationalization*

Individuals were geolocated to specific DeSO area in 2013 and followed up for the 3 years after. If an individual moved twice or more (between DeSO areas) during this period, they were classified as experiencing unstable housing.

*Numerator:* Individuals changing DeSO-area more than twice in the past three years

*Denominator:* Total population

*Age limits:* 18-100

*Geography:* DeSO

*Data sources:* LISA, GDB & RTB

**References**

1. SCB. Det Statistiska Registrets Framställning och Kvalitet: Registret över totalbefolkningen (RTB). Statistics Sweden (SCB); 2024.

2. Huisman M, Kunst AE, Bopp M, Borgan J-K, Borrell C, Costa G, et al. Educational inequalities in cause-specific mortality in middle-aged and older men and women in eight western European populations. The Lancet (British edition). 2005;365(9458):493-500.

3. Mackenbach JP, Kulhánová I, Bopp M, Deboosere P, Eikemo TA, Hoffmann R, et al. Variations in the relation between education and cause-specific mortality in 19 European populations: A test of the “fundamental causes” theory of social inequalities in health. Social science & medicine (1982). 2015;127(127):51-62.

4. Riddell WC, Song X. The impact of education on unemployment incidence and re-employment success: Evidence from the U.S. labour market. Labour economics. 2011;18(4):453-63.

5. Fella G, Gallipoli G. Education and Crime over the Life Cycle. The Review of economic studies. 2014;81(4 (289)):1484-517.

6. Willeck C, Mendelberg T. Education and Political Participation. Annual review of political science. 2022;25(1):89-110.

7. Oskarsson S, Dinesen PT, Dawes CT, Johannesson M, Magnusson PKE. Education and Social Trust: Testing a Causal Hypothesis Using the Discordant Twin Design. Political psychology. 2017;38(3):515-31.

8. Graham H. Understanding health inequalities. 2000.

9. Heckman J, Mosso, S. The Economics of Human Development and Social Mobility. Annual review of economics. 2014.

10. DeCicca P, Krashinsky H. The effect of education on overall fertility. Journal of population economics. 2023;36(1):471-503.

11. Falch T, Sandgren Massih S. The effect of education on cognitive ability. Economic inquiry. 2011;49(3):838-56.

12. Masters RK, Link BG, Phelan JC. Trends in education gradients of ‘preventable’ mortality: A test of fundamental cause theory. Social science & medicine. 2015;127:19-28.

13. Mirowsky J, Ross C. Education, learned effectiveness and health. London Review of Education. 2005;3:205-20.

14. Tamayo Martinez N, Xerxa Y, Law J, Serdarevic F, Jansen PW, Tiemeier H. Double advantage of parental education for child educational achievement: the role of parenting and child intelligence. European journal of public health. 2022;32(5):690-5.

15. El Nokali NE, Bachman HJ, Votruba-Drzal E. Parent Involvement and Children's Academic and Social Development in Elementary School. Child development. 2010;81(3):988-1005.

16. Ruiz M, Goldblatt P, Morrison J, Porta D, Forastiere F, Hryhorczuk D, et al. Impact of Low Maternal Education on Early Childhood Overweight and Obesity in Europe. Paediatric and perinatal epidemiology. 2016;30(3):274-84.

17. Balaj M, York HW, Sripada K, Besnier E, Vonen HD, Aravkin A, et al. Parental education and inequalities in child mortality: a global systematic review and meta-analysis. The Lancet (British edition). 2021;398(10300):608-20.

18. Moraeus L, Lissner L, Yngve A, Poortvliet E, Al-Ansari U, Sjöberg A. Multi-level influences on childhood obesity in Sweden: societal factors, parental determinants and child’s lifestyle. International Journal of Obesity. 2012;36(7):969-76.

19. Balaj M. Self-reported health and the social body. Social theory & health. 2022;20(1):71-89.

20. Fischer M, Karlsson M, Nilsson T. Effects of compulsory schooling on mortality: evidence from Sweden. International journal of environmental research and public health. 2013;10(8):3596-618.

21. Balaj M, Henson CA, Aronsson A, Aravkin A, Beck K, Degail C, et al. Effects of education on adult mortality: a global systematic review and meta-analysis. The Lancet Public health. 2024;9(3):e155-e65.

22. Beuermann DW, Bottan NL, Hoffmann B, Jackson CK, Vera-Cossio D. Does education prevent job loss during downturns? Evidence from exogenous school assignments and COVID-19 in Barbados. European economic review. 2024;162:104675.

23. Andersson B-E. Effects of Day-Care on Cognitive and Socioemotional Competence of Thirteen-Year-Old Swedish Schoolchildren. Child Development. 1992;63(1):20-36.

24. Datta Gupta N, Simonsen M. The effects of type of non-parental child care on pre-teen skills and risky behavior. Economics letters. 2012;116(3):622-5.

25. Barry KM, Avraam D, Cadman T, Elhakeem A, El Marroun H, Jansen PW, et al. Early childcare arrangements and children's internalizing and externalizing symptoms: an individual participant data meta-analysis of six prospective birth cohorts in Europe. The Lancet regional health Europe. 2024;45.

26. Dietrichson J, Lykke Kristiansen I, Viinholt BA. Universal preschool programs and long‐term child outcomes: a systematic review. Journal of economic surveys. 2020;34(5):1007-43.

27. Skolverket. Deltagande i Förskola. 2018.

28. Boverket/Delmos. Beskrivning av indikatorerna i Segregationsbarometern. Huddinge: Delegationen Mot Segregation; 2022.

29. Siegel M, Mielck A, Maier W. Individual Income, Area Deprivation, and Health: Do Income-Related Health Inequalities Vary by Small Area Deprivation? Health economics. 2015;24(11):1523-30.

30. EPRS. Poverty in the European Union: The crises and its aftermath. European Parliamentary Research Service; 2016.

31. Naijie G, Alessandra G, Patrick M, Fangzhou X, Hareth A-J. Financial stress and depression in adults: A systematic review. PloS one. 2022;17(2).

32. Kawachi I, Kennedy BP. Income inequality and health: pathways and mechanisms. Health services research. 1999;34(1 Pt 2):215-29.

33. Stromberg U, Baigi A, Holmen A, Parkes BL, Bonander C, Piel FB. A comparison of small-area deprivation indicators for public-health surveillance in Sweden. Scandinavian journal of public health. 2021:14034948211030353.

34. Merlo J, Ohlsson H, Chaix B, Lichtenstein P, Kawachi I, Subramanian SV. Revisiting causal neighborhood effects on individual ischemic heart disease risk: A quasi-experimental multilevel analysis among Swedish siblings. Social science & medicine. 2013;76(1):39-46.

35. Lee Y, Kemp PA, Reina VJ. Drivers of housing (un)affordability in the advanced economies: a review and new evidence. Housing studies. 2022;37(10):1739-52.

36. Robison LJ, Siles ME, Jin S. Social capital and the distribution of household income in the United States: 1980, 1990, and 2000. The Journal of socio-economics. 2011;40(5):538-47.

37. Jutz R. The role of income inequality and social policies on income-related health inequalities in Europe. International journal for equity in health. 2015;14(115):117-.

38. Spehar A. Navigating Institutions for Integration: Perceived Institutional Barriers of Access to the Labour Market among Refugee Women in Sweden. Journal of refugee studies. 2021;34(4):3907-25.

39. Wight V, Kaushal N, Waldfogel J, Garfinkel I. Understanding the link between poverty and food insecurity among children: Does the definition of poverty matter? Journal of children & poverty. 2014.

40. Cooper K, Stewart K. Does Household Income Affect children’s Outcomes? A Systematic Review of the Evidence. Child indicators research. 2021;14(3):981-1005.

41. Levesque AR, MacDonald S, Berg SA, Reka R. Assessing the Impact of Changes in Household Socioeconomic Status on the Health of Children and Adolescents: A Systematic Review. Adolescent research review. 2021;6(2):91-123.

42. Björkenstam E, Hjern A, Mittendorfer-Rutz E, Vinnerljung B, Hallqvist J, Ljung R. Multi-Exposure and Clustering of Adverse Childhood Experiences, Socioeconomic Differences and Psychotropic Medication in Young Adults. PloS one. 2013;8(1):e53551-e.

43. Yang-Huang J, van Grieken A, You Y, Jaddoe VWV, Steegers EA, Duijts L, et al. Changes in Family Poverty Status and Child Health. Pediatrics. 2021;147(4):1.

44. Richardson T, Elliott P, Roberts R. The relationship between personal unsecured debt and mental and physical health: A systematic review and meta-analysis. Clinical psychology review. 2013;33(8):1148-62.

45. Dackehag M, Ellegård L-M, Gerdtham U-G, Nilsson T. Debt and mental health: new insights about the relationship and the importance of the measure of mental health. European journal of public health. 2019;29(3):488-93.

46. Velez E, Cominole M, Bentz A. Debt burden after college: the effect of student loan debt on graduates' employment, additional schooling, family formation, and home ownership. Education economics. 2019;27(2):186-206.

47. Reeves A, McKee M, Mackenbach J, Whitehead M, Stuckler D. Public pensions and unmet medical need among older people: cross-national analysis of 16 European countries, 2004–2010. Journal of epidemiology and community health (1979). 2017;71(2):174-80.

48. Thornton M, Bowers K. Poverty in Older Adulthood: A Health and Social Crisis. Online journal of issues in nursing. 2024;29(1):1-12.

49. Barnay T. Health, work and working conditions: a review of the European economic literature. The European journal of health economics. 2016;17(6):693-709.

50. Matilla-Santander N, Muntaner C, Kreshpaj B, Gunn V, Jonsson J, Kokkinen L, et al. Trajectories of precarious employment and the risk of myocardial infarction and stroke among middle-aged workers in Sweden: A register-based cohort study. The Lancet regional health Europe. 2022;15:100314.

51. Koranyi I, Jonsson J, Rönnblad T, Stockfelt L, Bodin T. Precarious employment and occupational accidents and injuries - a systematic review. Scandinavian Journal of Work, Environment ＆ Health. 2018;44(4):341-50.

52. Hsu C-H, Engelhardt H. A Precarious Path to Partnership? The Moderating Effects of Labour Market Regulations on the Relationship Between Unstable Employment and Union Formation in Europe. European journal of population. 2024;40(1):12-.

53. Jaydarifard S, Smith SS, Mann D, Rossa KR, Nikooharf Salehi E, Gnani Srinivasan A, et al. Precarious employment and associated health and social consequences; a systematic review. Australian and New Zealand journal of public health. 2023;47(4):100074-.

54. SweJEM. SweJEM: Low Employment Quality [Available from: https://ki.se/en/imm/research/swejem-precarious-employment.

55. Ludvigsson JF, Svedberg P, Olén O, Bruze G, Neovius M. The longitudinal integrated database for health insurance and labour market studies (LISA) and its use in medical research. European journal of epidemiology. 2019;34(4):423-37.

56. Janlert U, Winefield AH, Hammarström A. Length of unemployment and health-related outcomes: a life-course analysis. European journal of public health. 2015;25(4):662-7.

57. Hensher M. Covid-19, unemployment, and health: time for deeper solutions? BMJ. 2020;371:m3687-m.

58. Dieckhoff M, Gash V. Unemployed and alone? Unemployment and social participation in Europe. International journal of sociology and social policy. 2015;35(1/2):67-90.

59. Bonoli G, Turtschi N. Inequality in social capital and labour market re-entry among unemployed people. Research in social stratification and mobility. 2015;42:87-95.

60. Lundin A, Lundberg I, Hallsten L, Ottosson J, Hemmingsson T. Unemployment and mortality—a longitudinal prospective study on selection and causation in 49321 Swedish middle-aged men. Journal of epidemiology and community health. 2010;64(1):22-8.

61. Mascherini M, Ledermaier S. Exploring the diversity of NEETs: Publications Office of the European Union Luxembourg; 2016.

62. Manhica H, Lundin A, Danielsson A-KJBo. Not in education, employment, or training (NEET) and risk of alcohol use disorder: a nationwide register-linkage study with 485 839 Swedish youths. 2019;9(10):e032888.

63. Benjet C, Hernández-Montoya D, Borges G, Méndez E, Medina-Mora ME, Aguilar-Gaxiola SJspdm. Youth who neither study nor work: mental health, education and employment. 2012;54:410-7.

64. Basta M, Karakonstantis S, Koutra K, Dafermos V, Papargiris A, Drakaki M, et al. NEET status among young Greeks: Association with mental health and substance use. 2019;253:210-7.

65. O'Higgins N, Brockie K. The Youth Guarantee, Vulnerability, and Social Exclusion Among NEETs in Southern Europe. Politics and governance. 2024.

66. Mawn L, Oliver EJ, Akhter N, Bambra CL, Torgerson C, Bridle C, et al. Are we failing young people not in employment, education or training (NEETs)? A systematic review and meta-analysis of re-engagement interventions. Systematic reviews. 2017;6(1):16-.

67. Gibson M, Petticrew M, Bambra C, Sowden AJ, Wright KE, Whitehead M. Housing and health inequalities: A synthesis of systematic reviews of interventions aimed at different pathways linking housing and health. Health & place. 2011;17(1):175-84.

68. Riksrevisionen. Trångboddhet – Konsekvenser för Hälsa och Skolresultat. Stockholm, Sweden; 2019.

69. Clair A, Baker E, Kumari M. Are housing circumstances associated with faster epigenetic ageing? Journal of epidemiology and community health (1979). 2024;78(1):40-6.

70. Hartig T, Fransson U. Housing tenure and early retirement for health reasons in Sweden. Scandinavian journal of public health. 2006;34(5):472-9.

71. Alm S, Bäckman O. ‘When it rains, it pours’: Housing evictions and criminal convictions in Sweden. European journal of criminology. 2022;19(4):612-31.

72. Makinde O, Björkqvist K, Österman K. Overcrowding as a risk factor for domestic violence and antisocial behaviour among adolescents in Ejigbo, Lagos, Nigeria. Global mental health. 2016;3:e16-e.

73. Boverket. Trångboddheten i Storstadsregionerna. Karlskrona; 2016. Contract No.: 2016:28.

74. Mann SL, Wadsworth ME, Colley JR. Accumulation of factors influencing respiratory illness in members of a national birth cohort and their offspring. Journal of epidemiology and community health (1979). 1992;46(3):286-92.

75. Aldridge RW, Pineo H, Fragaszy E, Eyre MT, Kovar J, Nguyen V, et al. Household overcrowding and risk of SARS-CoV-2: analysis of the Virus Watch prospective community cohort study in England and Wales. Wellcome open research. 2021;6:347-.

76. Gove WR, Hughes M, Galle OR. Overcrowding in the Home: An Empirical Investigation of Its Possible Pathological Consequences. Am Sociol Rev. 1979;44(1):59-80.

77. Wickes R, Zahnow R, Corcoran J, Hipp JR. Neighbourhood social conduits and resident social cohesion. Urban studies (Edinburgh, Scotland). 2019;56(1):226-48.

78. Boggess LN, Hipp JR. Violent Crime, Residential Instability and Mobility: Does the Relationship Differ in Minority Neighborhoods? Journal of quantitative criminology. 2010;26(3):351-70.

79. Auerswald CL, Lin JS, Parriott A. Six-year mortality in a street-recruited cohort of homeless youth in San Francisco, California. PeerJ (San Francisco, CA). 2016;4:e1909-e.

80. Tsai AC. Home foreclosure, health, and mental health: a systematic review of individual, aggregate, and contextual associations. PloS one. 2015;10(4):e0123182-e.

# Tables and figures

### Figure S1: IMDIS_O_ scores across all 5984 DeSO areas


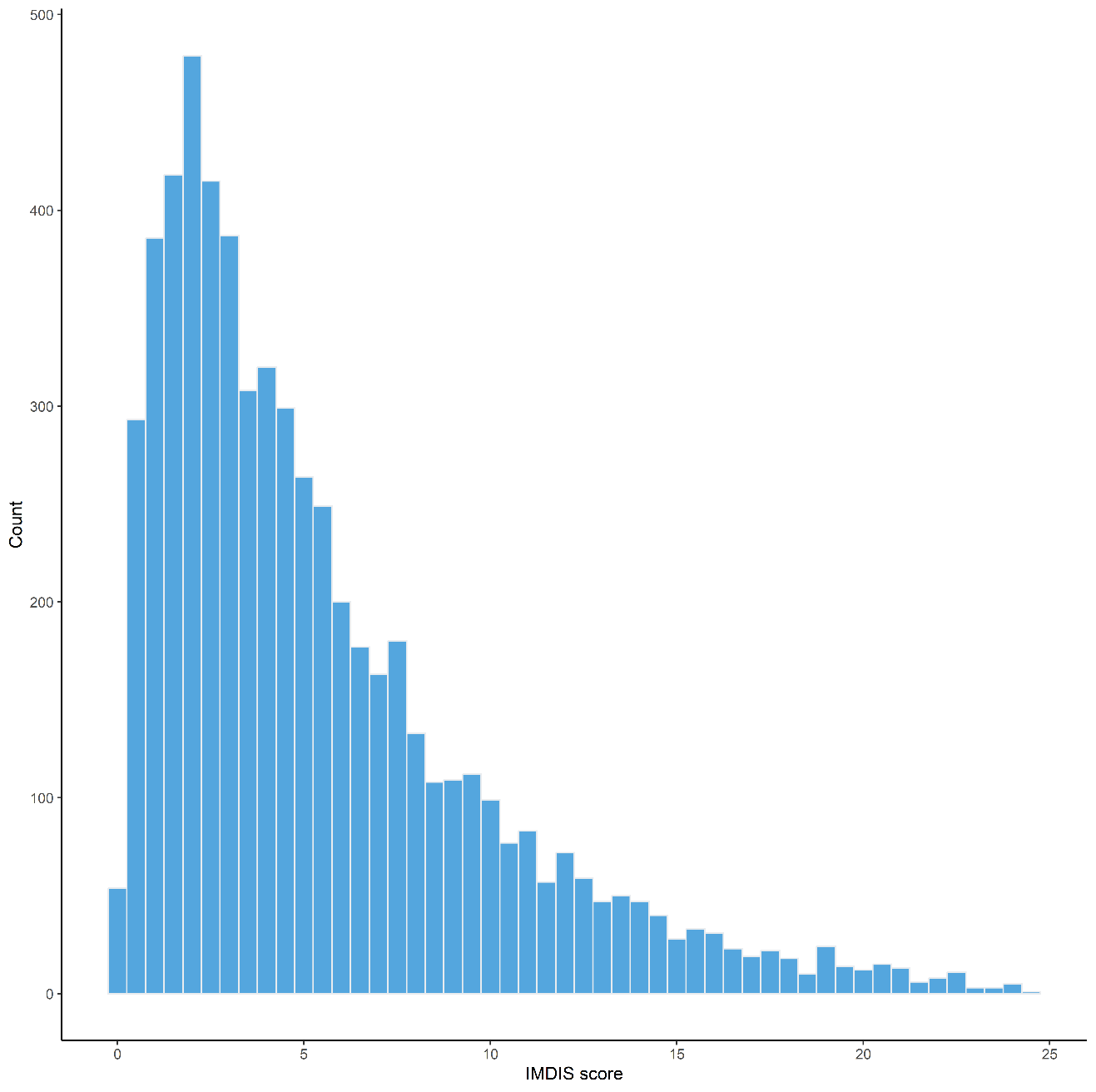


Figure S1: Histogram of the IMDIS_O_ scores across 5984 DeSO areas in Sweden 2015. A left skewed distribution with a right tail, illustrating the effect of the exponential transformation procedure.

### Figure S2: Correlation matrix of the IMDIS


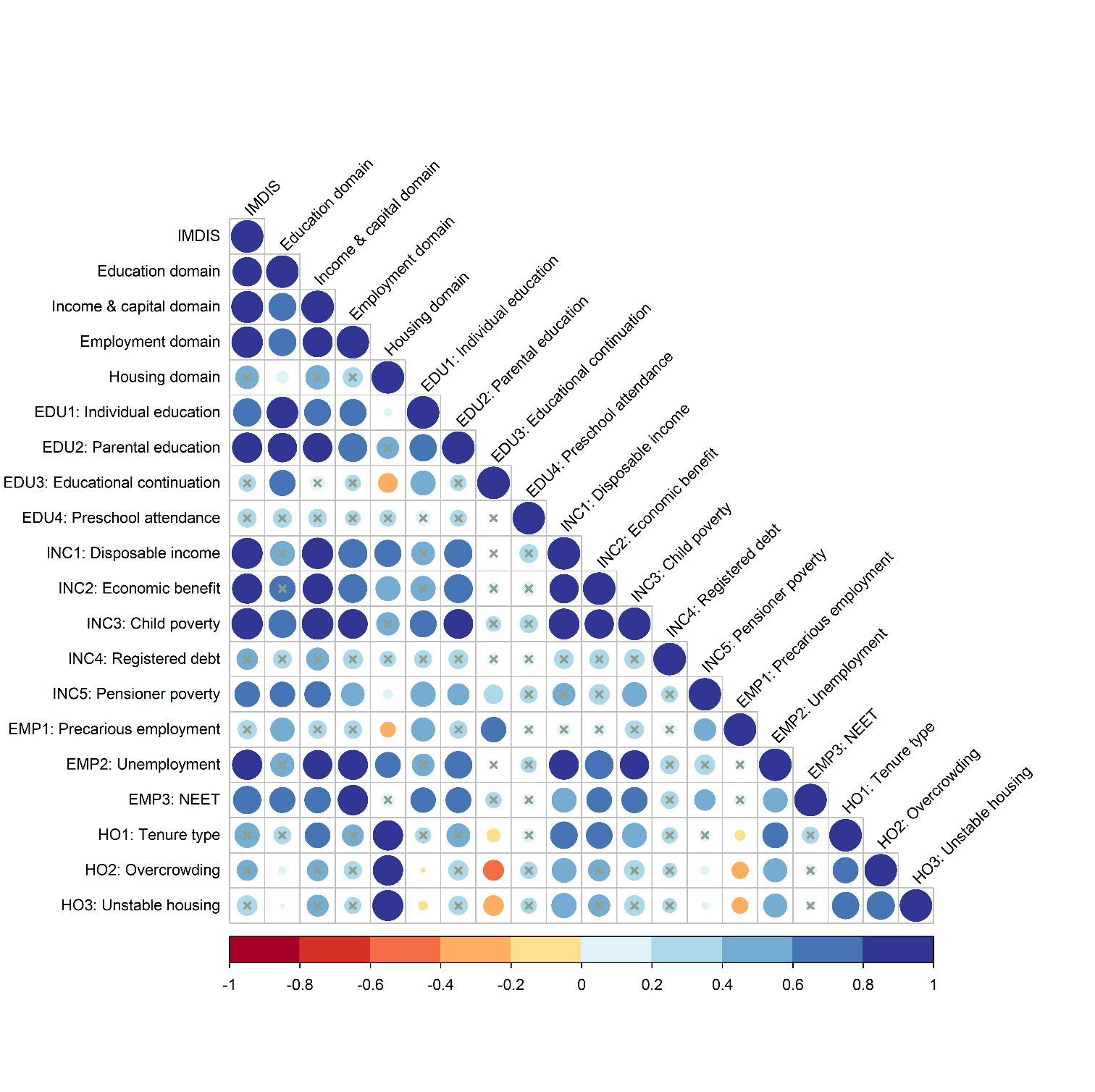


Figure S2: Spearman rank correlation matrix of the IMDIS, its domains and indicators. Non-significant coefficients (*p* > 0.05) are displayed with a light grey x.

### Figure S3: Distribution of IMDIS_O_ scores by 21 regions


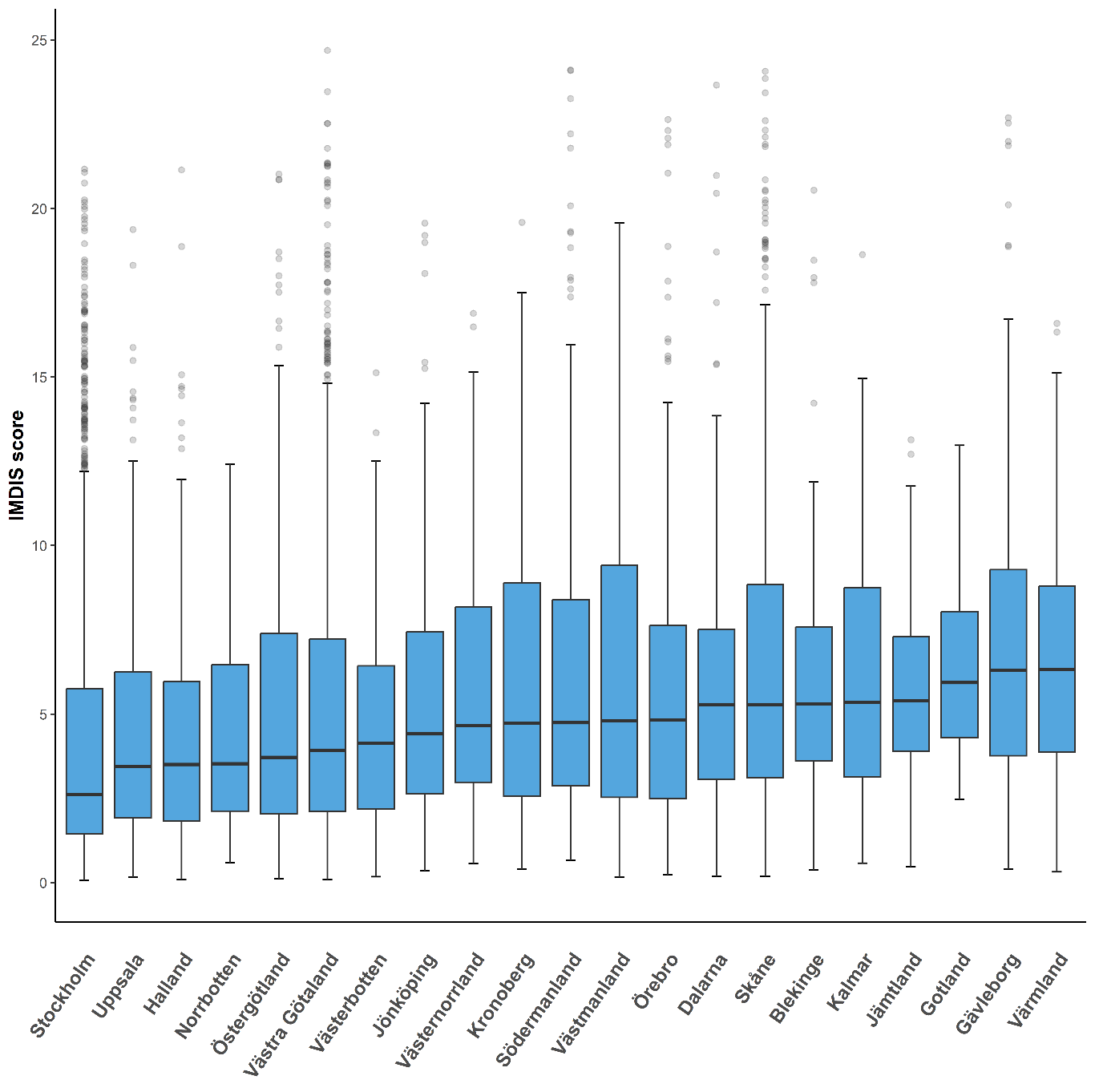


Figure S3: Boxplots of the IMDIS_O_ scores across 21 Swedish regions in 2015, with higher scores indicating more deprived areas.

###
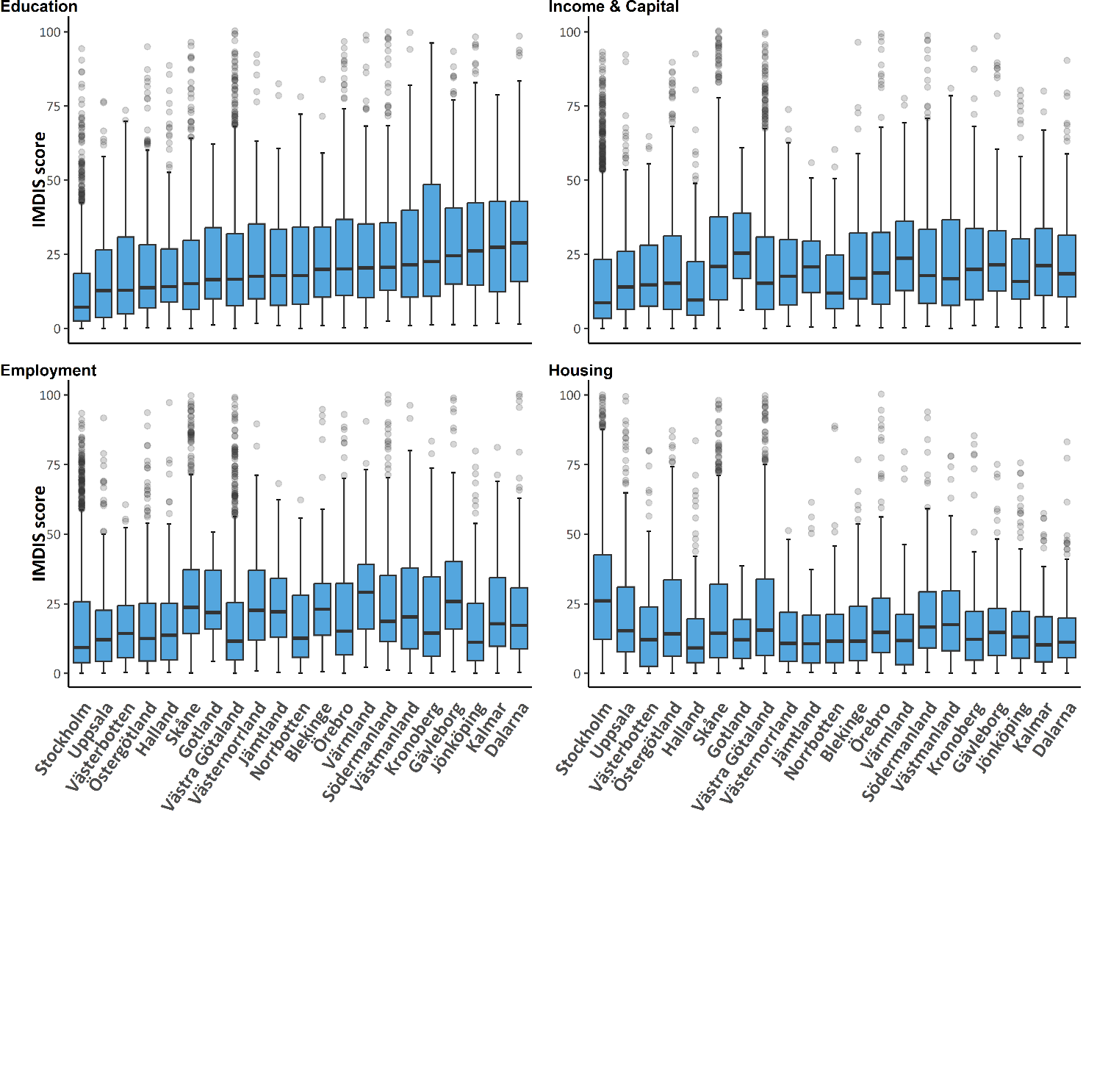
Figure S4: Distribution of domain-scores by region

Figure S4: Boxplots of domain specific scores across 21 Swedish regions in 2015, with higher scores indicating more deprived areas. Regions are sorted based on the distribution in the education domain.

### Table S1: Mean and total indicators scores, with numbers of DeSO areas, by population weighted deciles

|  |  | *Most deprived* | | | | **Deciles of deprivation** | | | | *Least deprived* | | | |
| --- | --- | --- | --- | --- | --- | --- | --- | --- | --- | --- | --- | --- | --- |
| **Indicator** | **Total** | **1^st^** | **2^nd^** | **3^rd^** | **4^th^** | | **5^th^** | **6^th^** | **7^th^** | | **8^th^** | **9^th^** | **10^th^** |
| EDU1: Individual education | 18.80 (5984) | 30.31 (538) | 25.24 (591) | 23.32 (614) | 21.03 (622) | | 19.9 (623) | 17.53 (611) | 15.74 (613) | | 13.9 (605) | 12.07 (600) | 9.68 (567) |
| EDU2: Parental education | 5.36 (5984) | 20.4 (538) | 10.28 (591) | 7.07 (614) | 4.78 (622) | | 3.71 (623) | 2.83 (611) | 2.28 (613) | | 1.75 (605) | 1.27 (600) | 0.82 (567) |
| EDU3: Educational continuation | 40.01 (5984) | 38.57 (538) | 44.23 (591) | 45.95 (614) | 45.27 (622) | | 43.6 (623) | 41.32 (611) | 38.94 (613) | | 36.64 (605) | 34.28 (600) | 30.25 (567) |
| EDU4: Preschool attendance | 18.27 (5984) | 22.16 (538) | 20.02 (591) | 18.94 (614) | 19.42 (622) | | 18.57 (623) | 18.3 (611) | 17.55 (613) | | 17.18 (605) | 16.42 (600) | 14.3 (567) |
| INC1: Disposable income | 19.01 (5984) | 7.01 (538) | 7.37 (591) | 7.55 (614) | 7.53 (622) | | 7.51 (623) | 7.31 (611) | 7.13 (613) | | 6.88 (605) | 6.57 (600) | 6.17 (567) |
| INC2: Economic benefit | 4.02 (5984) | 12.25 (538) | 7.18 (591) | 5.4 (614) | 4.5 (622) | | 3.96 (623) | 3.52 (611) | 3.2 (613) | | 2.9 (605) | 2.51 (600) | 2.05 (567) |
| INC3: Child poverty | 18.77 (5984) | 27.41 (538) | 20.34 (591) | 16.92 (614) | 14.92 (622) | | 13.66 (623) | 12.77 (611) | 11.59 (613) | | 11.17 (605) | 10.84 (600) | 10.38 (567) |
| INC4: Registered debt | 4.34 (5984) | 73.3 (538) | 46.26 (591) | 34.4 (614) | 29.56 (622) | | 24.6 (623) | 23.06 (611) | 21.67 (613) | | 17.32 (605) | 11.46 (600) | 3.96 (567) |
| INC5: Pensioner poverty | 5.91 (5984) | 21.67 (538) | 11.77 (591) | 8.75 (614) | 7.78 (622) | | 7.25 (623) | 7.88 (611) | 7.82 (613) | | 8.16 (605) | 7.57 (600) | 5.03 (567) |
| EMP1: Precarious employment | 7.11 (5984) | 10.01 (538) | 7.82 (591) | 6.61 (614) | 6.57 (622) | | 6.19 (623) | 6.32 (611) | 6.49 (613) | | 6.02 (605) | 5.4 (600) | 3.98 (567) |
| EMP2: Long-term unemployment | 4.67 (5984) | 45.5 (538) | 29.9 (591) | 23.65 (614) | 20.05 (622) | | 17.18 (623) | 15.57 (611) | 13.84 (613) | | 11.63 (605) | 9.18 (600) | 5.95 (567) |
| EMP3: Not in Employment, Education or Training (NEET) | 14.87 (5984) | 16.33 (538) | 7.22 (591) | 4.76 (614) | 3.49 (622) | | 2.69 (623) | 2.14 (611) | 1.73 (613) | | 1.41 (605) | 1.02 (600) | 0.72 (567) |
| HO1: Tenure type | 28.17 (5984) | 55.32 (538) | 34.08 (591) | 24.94 (614) | 19.91 (622) | | 15.98 (623) | 12.81 (611) | 10.48 (613) | | 8.08 (605) | 5.88 (600) | 3.7 (567) |
| HO2: Overcrowding | 9.24 (5984) | 9.02 (538) | 6.57 (591) | 5.43 (614) | 4.73 (622) | | 4.32 (623) | 4.03 (611) | 3.36 (613) | | 2.63 (605) | 2.19 (600) | 1.52 (567) |
| HO3: Unstable housing | 6.51 (5984) | 30.31 (538) | 25.24 (591) | 23.32 (614) | 21.03 (622) | | 19.9 (623) | 17.53 (611) | 15.74 (613) | | 13.9 (605) | 12.07 (600) | 9.68 (567) |

### Table S2: Average age with 95% Confidence intervals by population weighted deciles of IMDIS_O_ and level of urbanization

|  |  |  | *Most deprived* | | | | **Deciles of deprivation** | | | *Least deprived* | | |
| --- | --- | --- | --- | --- | --- | --- | --- | --- | --- | --- | --- | --- |
| **Domain** | **Urbanization** | **Total** | **1^st^** | **2^nd^** | **3^rd^** | **4^th^** | **5^th^** | **6^th^** | **7^th^** | **8^th^** | **9^th^** | **10^th^** |
| Average age (mean (95% CI)) | Overall | 40.96 (40.84 – 41.08) | 37.02 (36.61–37.43) | 41.49 (41.08–41.9) | 42.72 (42.35–43.09) | 42.51 (42.16–42.86) | 42.68 (42.35–43.01) | 41.77 (41.44–42.1) | 41.28 (40.97–41.59) | 41.05 (40.72–41.38) | 39.98 (39.67–40.29) | 38.41 (38.11–38.71) |
|  | Peripheral | 41.75 (41.44 – 42.06) | 43.28 (41.88–44.68) | 43.81 (43.13–44.49) | 43.39 (42.7–44.08) | 42.21 (41.36–43.06) | 42.25 (41.4–43.1) | 42.01 (40.85–43.17) | 41.32 (40.37–42.27) | 40.26 (39.43–41.09) | 40.16 (39.09–41.23) | 38.67 (37.85–39.49) |
|  | Rural | 43.76 (43.55 – 43.97) | 44.4 (39.67–49.13) | 46.92 (46–47.84) | 45.89 (45.41–46.37) | 45.18 (44.72–45.64) | 44.33 (43.9–44.76) | 42.81 (42.41–43.21) | 42.19 (41.72–42.66) | 41.58 (41.02–42.14) | 40.62 (39.82–41.42) | 41.49 (39.56–43.42) |
|  | Urban | 40.16 (40.02 – 40.30) | 36.53 (36.13–36.93) | 40.47 (40.02–40.92) | 41.55 (41.08–42.02) | 41.26 (40.79–41.73) | 41.85 (41.39–42.31) | 41.22 (40.76–41.68) | 40.95 (40.54–41.36) | 41.02 (40.62–41.42) | 39.87 (39.52–40.22) | 38.31 (37.98–38.64) |
